# Parkour as a Donor Sport for Athletic Development in Youth Team Sports: Insights Through an Ecological Dynamics Lens

**DOI:** 10.1186/s40798-018-0132-5

**Published:** 2018-05-24

**Authors:** Ben William Strafford, Pawel van der Steen, Keith Davids, Joseph Antony Stone

**Affiliations:** 1 Centre for Sports Engineering Research, Sheffield Hallam University, Broomgrove Teaching Block, Broomgrove Road, Sheffield, S10 2LX UK; 2 Academy of Sport and Physical Activity, Sheffield Hallam University, Collegiate Hall, Collegiate Crescent, Sheffield, S10 2BP UK

**Keywords:** Affordances, Athletic development, Athletic Skills Model, Donor sport, Early diversification

## Abstract

Analyses of talent development in sport have identified that skill can be enhanced through early and continued involvement in donor sports which share affordances (opportunities for action) with a performer’s main target sport. Aligning key ideas of the Athletic Skills Model and ecological dynamics theory, we propose how the sport of parkour could provide a representative and adaptive platform for developing athletic skill (e.g. coordination, timing, balance, agility, spatial awareness and muscular strength). We discuss how youth sport development programmes could be (re) designed to include parkour-style activities, in order to develop general athletic skills in affordance-rich environments. It is proposed that team sports development programmes could particularly benefit from parkour-style training since it is exploratory and adaptive nature shapes utilisation of affordances for innovative and autonomous performance by athletes. Early introduction to varied, relevant activities for development of athleticism and skill, in a diversified training programme, would provide impetus for a fundamental shift away from the early specialisation approach favoured by traditional theories of skill acquisition and expertise in sport.

## Key Points

- Traditional approaches to learning design that advocate early sport specialisation can hinder athletic development due to an overemphasis on the repetitive, drill-based nature of practice.
- Integrating parkour-style activities into practice could develop/maintain athleticism and promote skill transfer in an enjoyable environment in team sport athletes due to utilisation of performance-enhancing affordances and adaptive, functional, goal-directed movements.
- An ecological dynamic framework, in line with concepts from the Athletic Skills Model, has the potential to advance learning designs in sport based on commonality of affordances in parkour (as a donor sport) and team sports.

## Background

Expertise enhancement in sport requires athletes to engage in extensive training activities [1]. However, the types and amount of activities an athlete should engage with on the path to expertise has been debated [2]. Aligned with Ericsson’s [3] deliberate practice theory, emphasis on early specialisation has been typical with children encouraged to only practice and perform, from a very young age, in one target sport. To reach a high level of performance, early specialisation typically requires abundant coach-led, ‘deliberate practice’, designed to improve an athlete’s performance through frequent repetitions, use of instructions and frequent corrections, leading to movement reproduction [1, 4]. Early specialisation, however, can provide problems for a young athlete’s overall development, health and wellbeing, which can lead to a restricted range of general motor skills if children are only offered experience in a limited number of sports [5]. Focus on one sport can lead to increased risk of dropout due to lack of enjoyment in practising and competing in a fixed sport from a young age [6].

In contrast, an ecological dynamic perspective proposes a more balanced developmental experience, supporting early diversification which enables transfer of training from one sport context to another [7]. The value of diversified sport experiences is evident with many elite athletes having engaged in a range of sports during their development (often between 4 and 6 different sports) [1, 8]. Key theoretical ideas of ecological dynamics align with practitioner models of athlete development, such as the Athletic Skills Model (ASM), which presents a more nuanced approach to expertise attainment by advocating early and continued experiences in a diverse range of activities. This model of athlete development and performance proposes the transition of practice experiences from diversification to greater specialisation as athletes develop [9]. According to the ASM, to avoid the pitfalls of early specialisation, practice in specific youth sport programmes should be (re) designed to include experience of various physical activities, termed donor sports, which cultivate athletic skill development through exploratory practice and guided discovery. Here, we propose that parkour is an excellent candidate donor sport for developing talent in team games because athletes are challenged to negotiate obstacles of various textures, surfaces, inclination, area, sizes and angles to scale movement behaviours during interactions with their environment [10, 11]. These interactions are predicated on cognition, perception and action as athletes learn how to perform the same and different movements with respect to obstacle and surface properties. Parkour is unique compared to many other sports as the amount of traditional coaching is limited, with learning taking place via experience, observation and exploration rather than being driven by prescriptive coach-led instruction.

In this opinion piece, with specific reference to the tenets of the Athletic Skills Model [9], we outline how the relationship between task, environmental and organismic constraints and affordances [12], intrinsic to parkour activities and team sports, can be exploited, providing a conceptually and practically important means for designing programmes for athletic development and expertise enhancement in sport.

## Discussion

### The Athletic Skills Model and Donor Sports

**Fig. 1:**
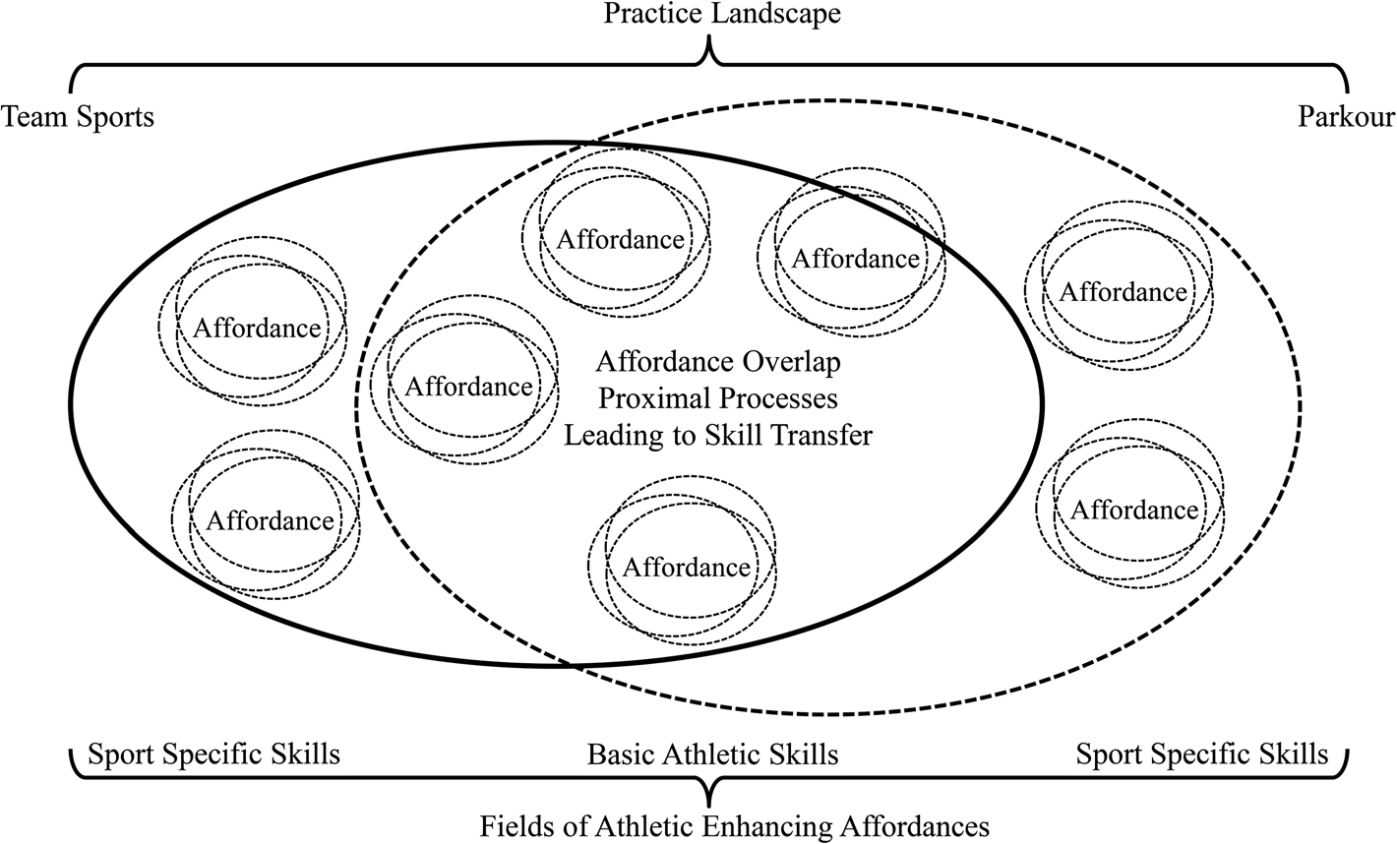
Overlap of performance-enhancing affordance fields between team sports and parkour as a donor sport

The ASM proposes that young athletes need to become versatile and adaptive movers, before they can become expert athletes (for detail overview of the ASM see Chapter 5 [9]). Two areas of the ASM, basic movement skills and donor sports, suggest that athletes, more generally, need to become attuned to affordances (opportunities for action [12]) in sport performance contexts. The ASM proposes that coaches need to design athlete development programmes that can enhance ten basic movement skills which can lead to diversified movement experiences to develop health, wellbeing and athletic potential [13]. Donor sports should promote transfer of varied and specific movement experiences across a range of non-specific and specific practice environments which support performance functionality at the moment of specialisation [14]. This approach to skill learning requires a careful and continuous transition between generality (non-target sports and activities) and specificity (engaging with various forms of a target sport) of transfer [15]. Practice tasks should develop general capacities that underpin functionality of each athlete’s current intrinsic dynamics and perceptual skills (e.g. anticipation, visual search, strength and postural stability) under a new set of performance constraints [16]. Insights of ecological dynamics suggest that donor sports and team sports share adjacent areas or fields of an *affordance landscape* [17] that include an extensive range of opportunities for action which can transfer functional performance behaviours from a donor sport to a target sport [9]. For example, in football, the side-step cutting manoeuvres needed when dribblers need to drive past opponents during a 1v1sub-phase of the game could be specifically transferred from performance of stepping and reaching actions in parkour. Transfer between the two sports is supported by the shared coordination dynamics intrinsic to these actions. These ideas on the transfer potential of overlapping fields in an affordance landscape (capturing relations between team sports and parkour) are depicted in Fig. 1.

Relevant, functional athletic abilities, including coordination, balance, turning ability, body awareness, strength and reaction speed can be augmented through interactions with an affordance landscape shared between a donor sport activity that supports exploratory learning and an athlete’s main target sport [9]. Donor sport activities should share many of the athletic movement functions required for performance in a target sport. But they can be particularly useful when a particular skill, relevant in a target sport, is considered to be under-developed in an athlete’s current repertoire [9]. This potential overlap of performance-enhancing affordances in the donor sport landscape presents opportunities for the development of athletic skills that require further development in an individual’s target sport (e.g. specific postural control and balance capabilities required for on and off the ball movement in soccer). These theoretical ideas provide a principled basis to help coaches understand how they can design training tasks to help athletes explore and exploit the complementarity between parkour and team sports. Additionally, participating in donor sports has potential psychological benefits such as enhanced perception, cognition and emotional self-regulation, as athletes begin to regulate anxiety during competition when they need to regulate their performance behaviours under pressure.

### Parkour as a Donor Sport

Parkour is an acrobatic sport where practitioners explore their action capabilities (typically running, climbing, jumping, bi-pedal or uni-pedal landing, hanging, vaulting, balancing, stepping, hurdling, quadrupedal movement and rolling) relative to their perceptual and motor abilities (coordination, timing, balance, agility, spatial awareness an muscular strength) to negotiate environmental features and properties (e.g. gaps, obstacles, surfaces and inclines) in the most innovative and efficient manner [11]. Performing these activities requires athletes to judge distances, gap sizes and surface properties and use cognitive skills such as perception, attention, problem-solving and creativity in negotiating environmental features. The challenges of a parkour learning environment can be (re) designed by manipulation of task, environment and organismic (personal) constraints. As athletes become more skilful at perceiving information from the environment, and coupling it to their actions, relative to their current athletic capabilities (known as effectivities), they will be able to transfer specific movements to performance in a target sport. A network of shared affordances in the environment can invite specific actions, which provide opportunities for athletic skill development as athletes become more adaptive at sampling a variety of environmental properties and energy flows intrinsic to parkour and a team sport being targeted [18]. Abilities deemed critical to athlete development can be ‘donated’ by parkour, such as fluidity of movement, safe landing strategies, creativity in negotiating gaps and obstacles and perception of information and related decision making. General transfer of training between parkour and team sports may be supported by learners being enabled to adapt their existing coordination dynamics. The coupling of perception and action, the fundamental basis of skilled behaviour in ecological dynamics, can be developed through exposure to parkour training to alter each athlete’s intrinsic dynamics (dispositions based on individual system development (puberty) status and skill level), which are shaped via continuous interactions of personal, task and environmental constraints.

Team sport athletes are required to dynamically re-organise movement system components relative to an opponent’s movements with respect to the ball position, direction and speed [19]. Parkour practitioners similarly emphasise the importance of fluidity and dynamism in movement exploration within a performance environment. One example is the precise foot placement required to negotiate constraints of a performance environment, such as the location and orientation of objects. Parkour practitioners target improvements in foot placement using striding techniques, during which athletes negotiate obstacles of various shapes and sizes to reach an intended location in the most efficient way, whilst maintaining movement at high speeds. Implementing such activities into the training and warm-up phase of contact team sports could develop foot placement and orientation with respect to a target location (e.g. a ball in space, field/court/surface markings or an opposing player).

Parkour can also help develop an athlete’s capacity to effectively use turning and cutting movements, suggested as a critical skill in team sports [20]. Parkour practitioners target improvements in turning ability and spatial awareness using the ‘tic tac’ technique, during which athletes have to approach obstacles and take off with a change of direction. The intention here is for the athlete to clear the obstacles or use perceptual variables, such as time to contact with the object, to regulate the next phase of movement [21]. A shared task goal in parkour and team sports is to react to perturbations in a performance environment, and the ‘tic tac’ activity could transfer between athlete development programmes. In team sports, this activity would target the compensatory athletic skills required during phase transitions where athletes couple their movements at various speeds relative to the movements of opponents, teammates and direction of the ball [8, 22]. The transfer of specific athletic skills using the affordance landscape is dependent on several factors such as the skill level, injury status and maturation of the athlete, and sport practitioners should adapt the difficulty of the activities during practice programmes accordingly.

Safe landing strategies as a means of recovering balance, initiating dynamic changes of direction, use of ‘soft feet’ in running and landing, and postural control following physical challenges (perturbations) are critical for players to avoid injuries [20, 23]. Parkour could help with developing targeted landing skills, by enhancing awareness of proprioceptive and haptic information from the soles of the feet and the lower limbs given that the ability to regain balance and postural control following physical challenges is continually required in parkour. In parkour, the roll landing strategy is explored during the early stages of learning, as the ability to land safely and then continue to move in a controlled manner, after experiencing a perturbation, is fundamental for an athlete’s performance, safety and wellbeing [24]. Parkour rolls appeared to be more appropriate (safer) for coaches to prescribe over traditional roll landing techniques, given the lower maximal vertical forces, slower times to maximal vertical force and ultimately lower loading rates [24]. Resourcefulness in movement exploration afforded through parkour would help athletes recover from forced landings in target sports such as rugby league and rugby union where players exert considerable force in tackles to regain ball possession [18, 25]. However, understanding of these implications of parkour training interventions for the development of safe landing techniques needs to be further developed in empirical research.

An important point is that parkour emphasises enjoyment and fosters creativity in movement exploration rather than focusing on developing movement skills in traditional drill-based repetitive practices. Emphasising enjoyment and creativity may reduce boredom and enhance movement coordination and control as every obstacle an athlete meets during parkour will need to be negotiated in a distinct way. Each interaction with a surface or an obstacle may not have an immediately obvious solution, so athletes must use their creativity to interact with them and solve performance problems in meaningful ways. Sport practitioners in youth team development programmes can exploit the exploratory and creative nature of parkour, to enable physical conditioning whilst at the same time enhancing perceptual, decision making and functionality of actions in an enjoyable way [26]. In youth team sport, a physical change in athletes during the adolescent growth spurt phases of puberty influences how affordances are perceived and acted upon. The playful nature of parkour activities would ensure that an athlete’s nervous system is adapted to coping with variations in cognitions, perceptions and actions that emerge during skill acquisition. These experiences afford an exploratory environment enabling performers to adapt to intrinsic changes during puberty (e.g. increased stature, rapid changes in limb properties, body mass and muscular strength) [9].

Parkour activities such as ‘follow the leader’ games, where groups of athletes copy each other’s movements, also have emotional benefits. The social dimension of these interactions with coaches and peers can help regulate athletes’ emotional control, resilience and self-confidence through a shared network of affordances rooted in a desire to interact with others and have fun. The playful aspects of parkour allow athletes to explore and consolidate movement patterns they may normally avoid. This implicit focus of skill learning educates the athletes’ attention towards the performance environment, supporting affordance selection and utilisation through intrinsic capacities, described as *effectivities* [27–29]. Individuals trained in parkour may perceive and interact with the surrounding environment and task context, with an adaptive behavioural flexibility for the discovery of athletic enhancing affordances that other athletes not trained in parkour cannot specify [30]. Moreover, parkour athletes demonstrate the capacity to manage risk in their environmental interactions, skilfully and creatively. In this way, exposure to parkour-style activities could help youth athletes learn to undertake risk-benefit analyses in contact team sports, on and off field. Additionally, regular implicit practice in the playful and exploratory learning environments afforded by parkour could help regulate stress, reduce performance anxiety and increase resilience as athletes can become more proficient at utilising affordances with their athletic capabilities.

### Implications for Learning Design

Popular media can portray parkour as an extreme sport consisting of only large-scale movements that are of high injury risk, such as jumping from buildings or between train carriages. Whilst affordances present in some urban and forest environments offer opportunities to train parkour, the premise that children should be protected from potential injuries during play has resulted in the design of symmetrical and standardised children’s playgrounds. These learning environments are indeed safe, but lack the variability required to satisfy the athletic developmental needs of child, such as capacities to make decisions quickly and land safely during force landings, common in team sports [31]. The rise in popularity of parkour globally has led to a development of dedicated indoor and outdoor parkour facilities where people can train [31, 32]. These parks represent a fundamental shift in playground design as learning environments which include design features, limitations or boundaries that constrain the (re) organisation of motor system degrees of freedom at different levels [33–35]. Parkour activities promote implicit skill acquisition, given that the performance environment is ever changing and variable, even in custom built parkour playgrounds. Therefore, where financial constraints are not an issue, academies of sports organisations should construct or regularly visit a parkour training facility, given that coaches can control the variability afforded in the parkour landscape [9]. For example, the use of landing mats should be prohibited during parkour training, as this will facilitate young athletes’ awareness of risk relative to their own abilities; allowing them to consider their current intrinsic dynamics or effectivities during movement exploration.

Despite the advantages afforded through interaction with a parkour landscape, the design and construction of these environments can be expensive. Parkour actions can also emerge from basic athletic skills that an athlete can undertake in affordance landscapes which do not require specialist equipment. Therefore, where visiting/constructing a parkour playground is not possible, learning practitioners could integrate parkour-style activities into regular training sessions as a means of presenting athletic enhancing affordances and problem-solving opportunities for athletes. These environments would include a scaled-down version of parkour-style activities relative to a performers’ age, skill level and the athletic skills being targeted. Equipment demands could include a flat surface (perhaps varying in texture), ledges, gaps, obstacles, other performers, cones, inclines and surface markings. This equipment would allow athletes to selectively engage with a landscape of performance-enhancing affordances and explore their athletic movement capacities, shared with performance behaviours of a target sport [36, 37]. Further, the exploratory nature of these activities will engage students to play games and face challenges together to stimulate self-organisation, discovery learning, exploratory activities and improved emotional self-regulation. Involving participants in game design is appropriate for cognitive engagement and the development of awareness and social interactions.

Aligned with the main tenets of the AMS model, in designing these activities, sport practitioners should limit explicit feedback, correction and instruction during parkour-style practice, as task constraint configurations do not prescribe each learner’s emergent behaviours, but simply guide them implicitly [9, 38]. Depending on a performer’s age, ability and skill level, the practitioner can also explicitly guide learners towards functional behaviours, if needed. However, feedback and instruction should be provided to help children to reflect, so that they can learn to adapt the functionality of their perception, cognitions and actions [9].

## Conclusions

Parkour provides a novel and representative adaptive performance platform for guiding learners through all stages of athletic development. A parkour-style training environment can be created that provides tasks to develop specific athletic abilities required in team sports, such as coordination, balance, agility, changing direction, controlled landing with ‘soft feet’, spatial awareness and strength of the entire body. The ASM, in line with concepts in ecological dynamics, advances learning designs in sport based on the commonality of perceptual, cognitive and actions demands (affordances) of donor sports (e.g. parkour) and target sports (e.g. team sports). Parkour-style training can provide a vehicle for athletic development and skill transfer through exploiting guided discovery learning in diversified sport experiences and environments. This approach would counteract the negative effects of early specialisation which can sometimes be tedious, too repetitive and drill-based, lacking intensity and dynamism in practice.

## Abbreviation

ASM: Athletic Skills Model
